# Bioinformatic Analysis of Circular RNA-Associated ceRNA Network Associated with Hepatocellular Carcinoma

**DOI:** 10.1155/2019/8308694

**Published:** 2019-11-03

**Authors:** Jiacheng Wu, Shui Liu, Yien Xiang, Xianzhi Qu, Yingjun Xie, Xuewen Zhang

**Affiliations:** ^1^Department of Hepatobiliary and Pancreatic Surgery, Second Hospital of Jilin University, Changchun 130041, China; ^2^Jilin Engineering Laboratory for Translational Medicine of Hepatobiliary and Pancreatic Diseases, Changchun 130041, China

## Abstract

Hepatocellular carcinoma (HCC) is the sixth most common cancer worldwide and is associated with a high mortality rate and poor treatment efficacy. In an attempt to investigate the mechanisms involved in the pathogenesis of HCC, bioinformatic analysis and validation by qRT-PCR were performed. Three circRNA GEO datasets and one miRNA GEO dataset were selected for this purpose. Upon combined biological prediction, a total of 11 differentially expressed circRNAs, 15 differentially expressed miRNAs, and 560 target genes were screened to construct a circRNA-related ceRNA network. GO analysis and KEGG pathway analysis were performed for the 560 target genes. To further screen key genes, a protein-protein interaction network of the target genes was constructed using STRING, and the genes and modules with higher degree were identified by MCODE and CytoHubba plugins of Cytoscape. Subsequently, a module was screened out and subjected to GO enrichment analysis and KEGG pathway analysis. This module included eight genes, which were further screened using TCGA. Finally, UBE2L3 was selected as a key gene and the hsa_circ_0009910–miR-1261–UBE2L3 regulatory axis was established. The relative expression of the regulatory axis members was confirmed by qRT-PCR in 30 pairs of samples, including HCC tissues and adjacent nontumor tissues. The results suggested that hsa_circ_0009910, which was upregulated in HCC tissues, participates in the pathogenesis of HCC by acting as a sponge of miR-1261 to regulate the expression of UBE2L3. Overall, this study provides support for the possible mechanisms of progression in HCC.

## 1. Introduction

Hepatocellular carcinoma (HCC) is one of the most common malignancies of the digestive system, with a high mortality rate making it the sixth leading cause of cancer-related mortality worldwide [1, 2]. More than 466,100 new cases of HCC and 426,100 deaths occurred in China each year due to HCC [3]. A research focus has been placed on how to improve the prognosis of HCC patients. In recent years, the role of noncoding RNA in the development and progression of tumors has gradually been recognized by researchers in line with the deepening of research on the molecular mechanisms associated with tumors. The main categories of noncoding RNA are microRNA (miRNA), long noncoding RNA (lncRNA), and circular RNA (circRNA). CircRNA is a special kind of endogenous noncoding RNA that is widely present in all organs and tissues; it was first identified in viruses in 1970 [4–7]. More than a million different circRNAs have since been identified in human tissue by high-throughput sequencing [8]. Some studies have also confirmed that circRNAs could potentially serve as molecular markers or therapeutic targets for certain diseases, particularly in cancer growth, metastasis, and therapy resistance [9–12]. Although a comprehensive understanding of the functions of circRNAs has not been obtained, recent studies have shown that they function as microRNA sponges via competitive binding to the microRNA response element (MRE) to regulate the expression of target genes of microRNAs [5, 13]. Many studies have also shown that circRNAs play important roles in the development of HCC. For example, a study by Han et al. found that circMTO1 could suppress HCC progression by acting as a sponge of oncogenic miR-9 to promote p21 expression. Another study by Yu et al. found that Cirs-7 competitively bound to miR-7 to derepress the expression of CCNE1 and PIK3CD genes to promote the proliferation and invasiveness of liver cancer cells [14, 15]. Nonetheless, more research is still needed to explore the roles of circRNAs in the pathogenesis of HCC.

**Figure 1:**
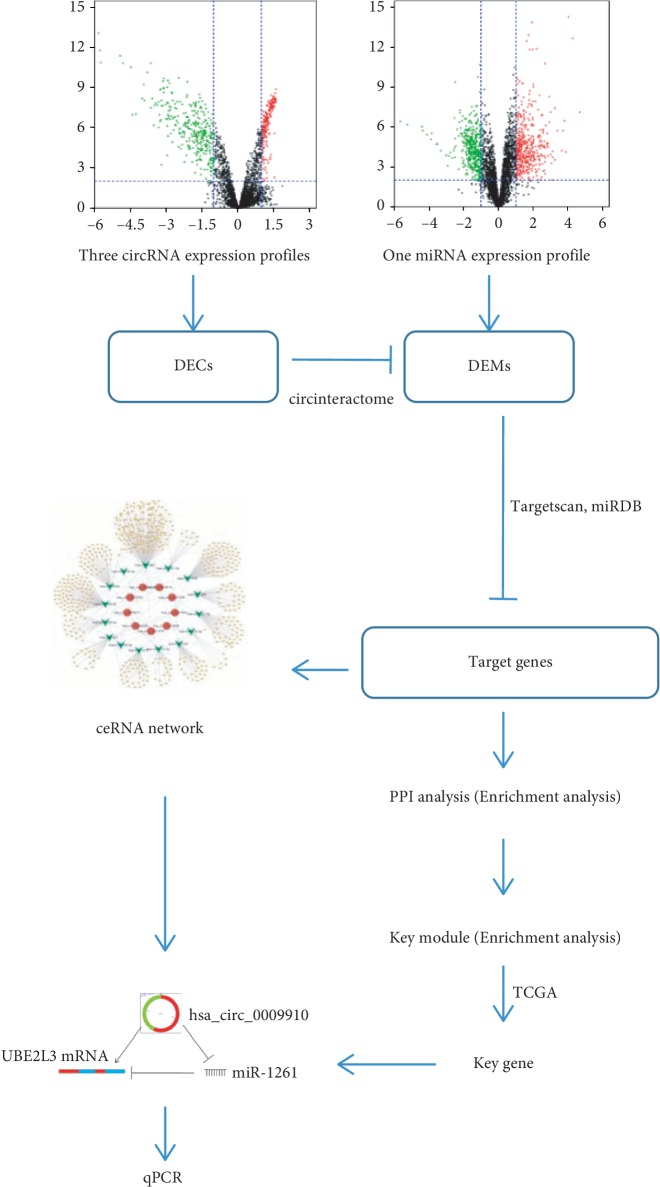
Process of circRNA-related ceRNA regulatory network construction and identification of key genes in HCC.

Considering that the circRNAs and microRNAs differentially expressed between hepatocellular carcinoma tissues and adjacent noncarcinoma tissue may play important roles in HCC development and progression, three circRNA expression profiles (GSE78520, GSE97332, and GSE94508) and one miRNA expression profile (GSE64632) were downloaded from the Gene Expression Omnibus (GEO) database of the National Center of Biotechnology Information to obtain differentially expressed circRNAs (DECs) and differentially expressed miRNAs (DEMs) using R software. The interactions of circRNA and miRNA, and miRNA and mRNA were predicted using online databases, and 560 target genes were obtained. Subsequently, Gene Ontology (GO), Kyoto Encyclopedia of Genes and Genomes (KEGG) pathway enrichment, and protein-protein interaction (PPI) network analyses were performed to reveal the interactive relationships among the target genes to explore the underlying molecular mechanisms involved in the carcinogenesis and progression of HCC [16, 17]. A gene module was screened from the PPI network and further validated for the expression levels of the genes and clinical relevance using The Cancer Genome Atlas (TCGA). Finally, UBE2L3 was screened out and its ceRNA regulatory network was constructed; the expression levels of molecules in this regulatory network were also confirmed by qPCR in tumor tissues and adjacent nontumor tissues. The purpose of this study was to provide valuable insights for biomarker discovery and the development of a novel treatment strategy for HCC (Figure 1).

## 2. Materials and Methods

### 2.1. Microarray Data

Microarray datasets providing circRNA and miRNA expression profiles of HCC were downloaded from the Gene Expression Omnibus (GEO) database [18]. The three circRNA expression profiles (GSE78520, GSE97332 [19], and GSE94508 [20]) were from the platform of GLP19978. A total of 15 pairs of samples of HCC tissues and adjacent nontumor tissues were included in the circRNA microarray dataset. The miRNA expression profile of GSE64632 [21] was from the platform of GPL18116, which contained three pairs of samples of HCC tissues and adjacent nontumor tissues (Table 1).

### 2.2. Differential Expression Analysis

The “limma” package (3.38.3) in R (5.3.2) was applied to screen differentially expressed circRNAs (DECs) and differentially expressed miRNAs (DEMs) between HCC samples and adjacent nontumor samples. The significantly DECs (*P* < 0.01 and FC > 2) of each of the three circRNA expression profiles were identified. The overlapping upregulated and downregulated DECs were analyzed using FunRich software (available online: http://www.funrich.org/). The thresholds of *P* < 0.05 and FC > 2 were set to screen the significantly DEMs from the miRNA expression profile.

### 2.3. ceRNA Analysis of circRNA-Related Genes

The Circular RNA Interactome (https://circinteractome.nia.nih.gov/) was used to predict miRNA binding sites (MREs), excluding context score percentile lower than 75, which were considered as potential target miRNAs of the DECs. These target miRNAs of DECs were further screened by overlapping with the DEMs identified previously. The interactions of miRNAs and mRNAs were established using TargetScan (http://www.targetscan.org) and miRDB (http://www.mirdb.org). We identified mRNAs overlapping between the two algorithms as potential target genes of the miRNAs. The interactive networks of DEGs, DEMs, and target genes were thus established and visualized using Cytoscape 3.6.1.

### 2.4. Functional Enrichment Analysis of Target Genes

Gene Ontology (GO) annotation and Kyoto Encyclopedia of Genes and Genomes (KEGG) pathway analysis of the target genes were carried out using Omicsbean online database (http://www.omicsbean.cn/). *P* < 0.05 was considered statistically significant.

### 2.5. PPI of Target Genes and Identification of Key Module

A protein-protein interaction (PPI) network of the target genes was established using STRING (v10.5) [22]; the minimum required interaction score was set to 0.4 and the network was visualized with Cytoscape 3.6.1. CytoHubba, a plugin in Cytoscape, was used to identify hub genes of the PPI network. “Molecular Complex Detection” (MCODE), another plugin of Cytoscape, was used to analyze modules of the PPI network, with the degree cut-off set to 5. The key module was identified and combined hub genes and modules GO analysis and KEGG pathway analysis were also performed on this key module.

### 2.6. Further Screening of Module Genes by TCGA

Screening of the module genes in TCGA dataset was performed by GETIA (http://gepia.cancer-pku.cn/) [23]. Expression analysis for different sample types (HCC and normal liver tissues), association analysis of gene expression level and LIHC patients' tumor stages, and analyses of overall survival and disease-free survival were also performed.

### 2.7. Quantitative Real-Time PCR Analysis

A total of 30 HCC patients were recruited for the sampling of tumor and adjacent nontumor tissues. This study was approved by the Ethics Committee of the Second Clinical Medical College, Jilin University. Total RNA extraction was performed using Trizol reagent (Sangon Biotech, Shanghai, China). Reverse transcription of circRNA and mRNA was performed using First Strand cDNA Synthesis Kit (Sangon Biotech), and reverse transcription of miRNA was performed using miRNA First Strand cDNA Synthesis (tailing reaction) (Sangon Biotech), with the following primers: hsa_circ_0009910 (forward: 5′-GCAGAACTGGACCCCGTTACC-3′, reverse: 5′-CAGGGACATTGCGCGGCCAA-3′), UBE2L3 (forward: 5′-TTAGTGCCGAAAACTGGAAGC-3′, reverse: 5′-ATTCACCAGTGCTATGAGGGAC-3′), and GAPDH (forward: 5′-GACAGTCAGCCGCATCTTCT-3′, reverse: 5′-ACCAAATCCGTTGACTCCGA-3′) synthesized by Sangon Biotech. Primers of miR-1261 and U6 were purchased from GeneCopoeia (Guangzhou, China). The primer specificity of hsa_circ_0009910 was verified by Circprimer1.2.0.5 [24] and Sanger sequencing. Real-time PCR was performed using 2×SG Fast qPCR Master Mix (Sangon, China), in accordance with the manufacturer's protocol, and the LightCycler 480II Fast Real-Time PCR System was applied (Roche, Indianapolis, IN, USA). All procedures were performed in accordance with the manufacturers' protocols. The relative expression was normalized to GAPDH and/or U6 expression by the comparative CT method. The relative expression was calculated using the 2^−ΔΔCT^ method.

## 3. Result

### 3.1. DECs Based on Three Microarray Datasets

**Figure 2:**
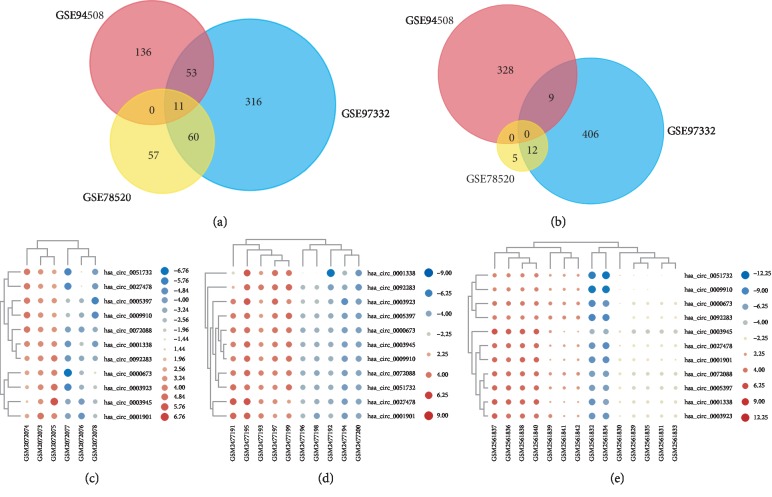
Differentially expressed circRNA screening. (a) Venn analysis of commonly upregulated circRNAs. (b) Venn analysis of commonly downregulated circRNAs. Heatmap of 11 commonly upregulated circRNAs in GSE78520 (c), GSE97332 (d), and GSE94508 (e).

Three microarray databases (GSE78520, GSE97332, and GSE94508) were included in our study, all of which were from the same platform of GPL19978. A summary of the three databases is presented in Table 1. Based on the thresholds of *P* < 0.01 and |LogFC| > 1, a total of 145 DECs (128 upregulated and 17 downregulated) were identified in profile GSE78520, 867 DECs (440 upregulated and 427 downregulated) in profile GSE97332, and 537 DECs (200 upregulated and 337 downregulated) in profile GSE94508 (Table 2). Eleven co-upregulated DECs (hsa_circ_0072088, hsa_circ_0051732, hsa_circ_0005397, hsa_circ_0000673, hsa_circ_0001338, hsa_circ_0003945, hsa_circ_0027478, hsa_circ_0092283, hsa_circ_0003923, hsa_circ_0009910, and hsa_circ_0001901) were identified by Venn analysis among the three databases, while no co-downregulated DECs were identified (Figure 2). The basic characteristics of the differentially expressed circRNAs are shown in Table 3.

### 3.2. Construction of the ceRNA Network

**Figure 3:**
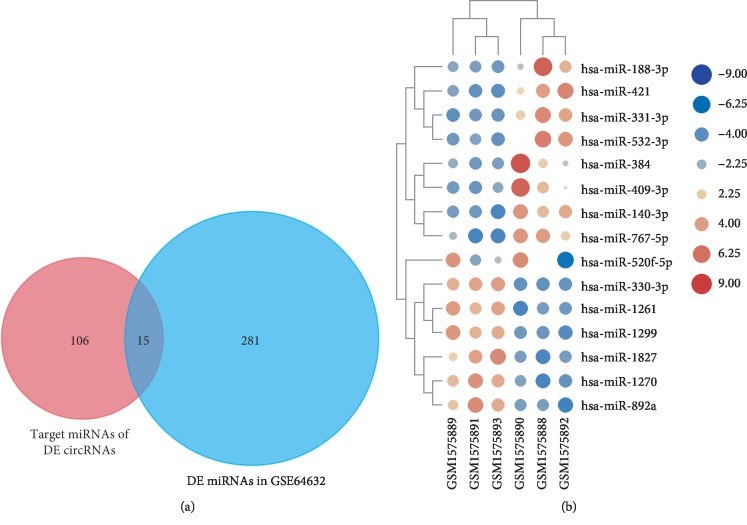
Identification of differentially expressed miRNAs. (a) Venn analysis of target miRNAs of DECs and differentially expressed miRNAs of GSE64632 dataset. (b) Heat map of 15 DEMs in GSE64632 dataset.

**Figure 4:**
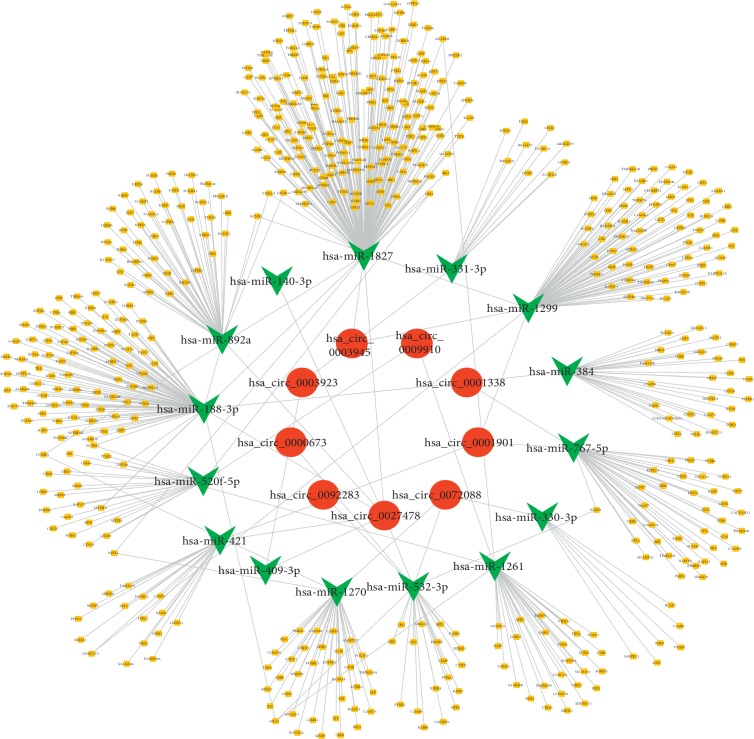
The network of circRNA-miRNA-target genes. Red represents circRNA, green represents miRNA, and yellow represents target gene.

A total of 11 circRNAs were selected for further study, for which a total of 180 target miRNAs were predicted by CircInteractome. Based on the thresholds of *P* < 0.05 and |LogFC| > 1, a total of 315 differentially expressed miRNAs were screened out in GSE64632, including 227 upregulated and 88 downregulated ones (Table 2). Fifteen DEMs were identified by Venn analysis among the 180 target miRNAs and 296 differentially expressed miRNAs, including 8 with upregulated expression and 7 with downregulated expression (Figure 3). The target genes for each of these 15 DEMs were predicted using two online databases, TargetScan and miRDB, and a total of 560 mRNAs that overlapped between the two databases were selected as target genes. The circRNA-miRNA-target gene network is shown in Figure 4.

### 3.3. Enrichment Analysis of the Target Genes

**Figure 5:**
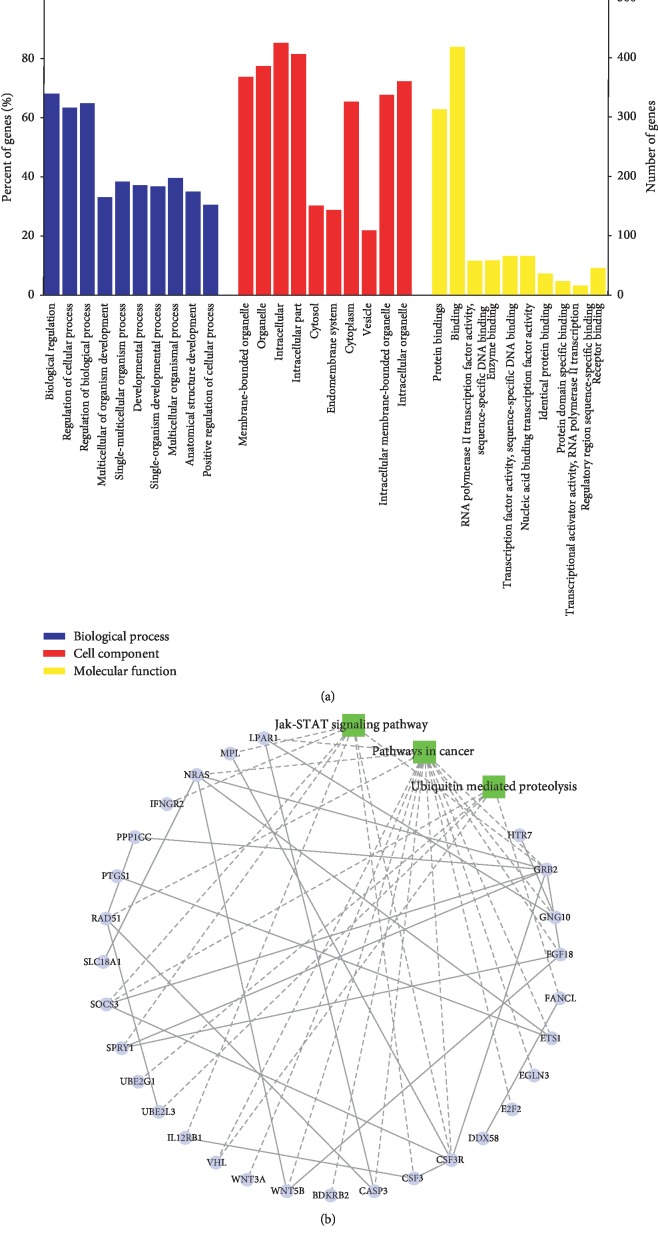
Enrichment analysis of target genes. (a) GO enrichment analysis of target genes. (b) KEGG pathway analysis of target genes.

GO and KEGG enrichment analyses were performed for the 560 target genes of the DEMs to investigate the biological functions of the circRNAs. The top 10 GO terms of each group are shown in Figure 5(a). In the biological process category, the main enriched categories were “biological regulation,” “regulation of cellular process,” and “regulation of biological process.” In the cellular component category, the main enriched categories were “membrane-bounded organelle,” “organelle,” and “intracellular.” In the molecular function category, the main enriched categories were “protein binding,” “binding,” and “RNA polymerase II transcription factor activity, sequence-specific DNA binding.” Finally, in the KEGG pathway analysis, the most enriched KEGG pathways were “ubiquitin-mediated proteolysis,” “JAK-STAT signaling pathway,” and “pathway in cancer.” The top 3 enriched cancer-related pathways are shown in Figure 5(b).

### 3.4. Identification of Key Module in the PPI Network

**Figure 6:**
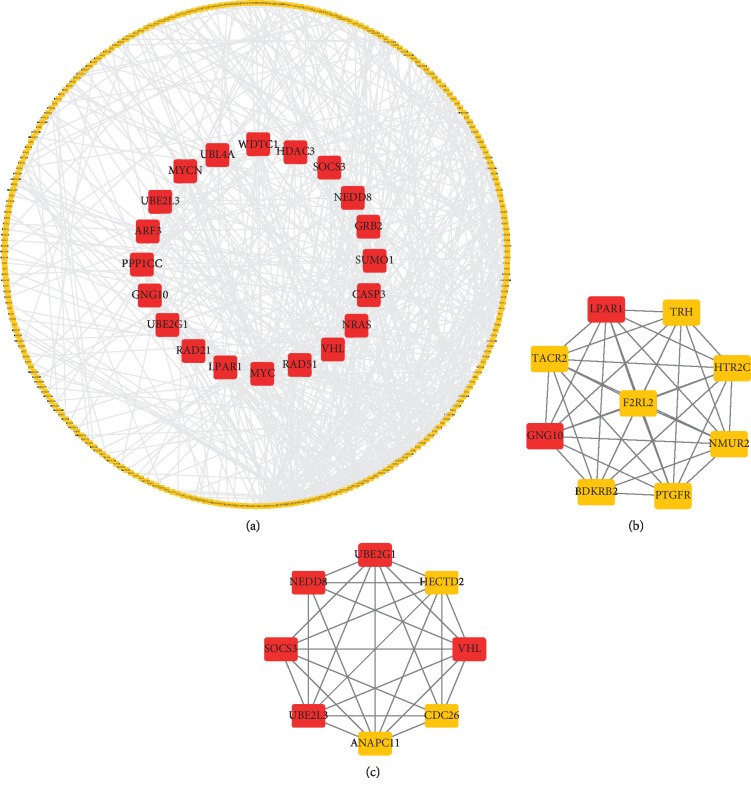
PPI analysis. (a) PPI analysis and hub gene screening of target genes. (b) Module 1 of the PPI network. (c) Module 2 of the PPI network. Red represents hub genes.

On the basis of the STRING database, we established a PPI network to show the interactions of the 560 target genes, and the top 20 hub genes were selected using CytoHubba, which were the nodes with higher degree in the network. Further MCODE analysis revealed two modules from the network. Module 1 consisted of 9 genes and 72 edges, while module 2 consisted of 8 genes and 63 edges. We found that 5 of the 8 genes in module 2 were hub genes of the PPI network, while only 2 of the 9 genes were in module 1. Therefore, we identified module 2 as the key module for further analysis. The PPI network, hub genes, and modules are shown in Figure 6.

### 3.5. Enrichment Analysis of Key Module

**Figure 7:**
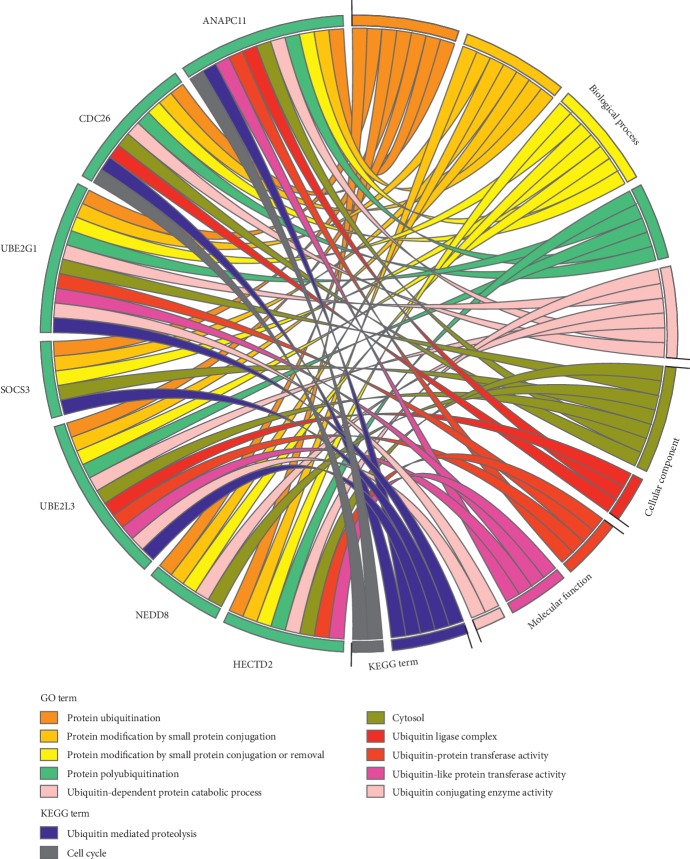
Tumor-related GO enrichment analysis and KEGG pathway analysis of key module genes.

To further explore the biological function of the key module, functional enrichment analysis was performed based on the Omicsbean database. Regarding the GO terms, the main enriched ones were protein ubiquitination, cytosol, ubiquitin ligase complex, and ubiquitin-protein transferase activity. The KEGG signaling pathway analysis showed marked enrichment of ubiquitin-mediated proteolysis. The results are shown in Figure 7.

### 3.6. TCGA Database Analysis

**Figure 8:**
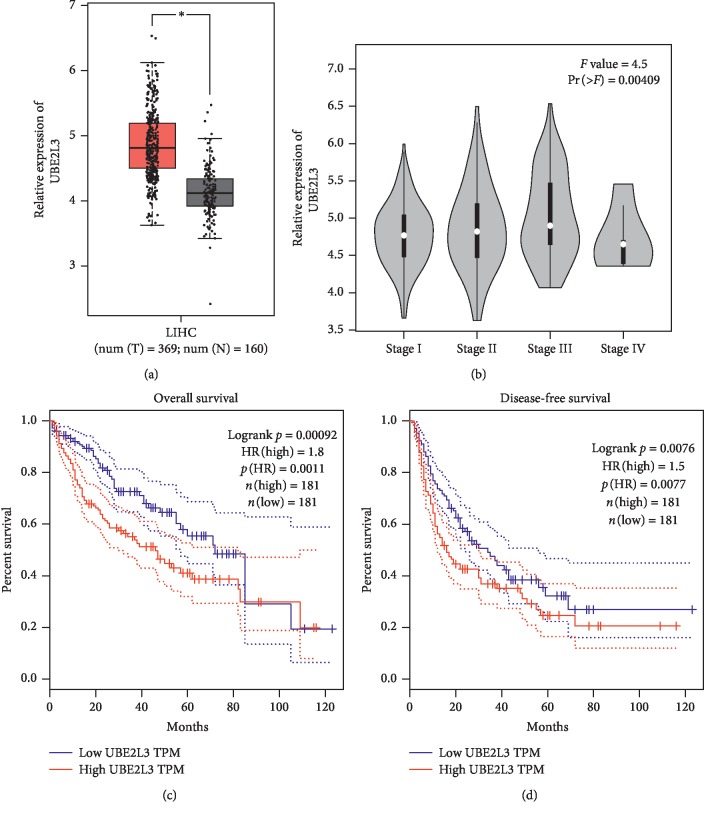
TCGA analysis of UBE2L3 by GEPIA. (a) Boxplots depicting expression levels in HCC versus nontumor liver tissues. (b) Violin plots depicting expression levels associated with tumor grades in HCC. (c) Kaplan–Meier plots comparing the overall survival rates with high expression and low expression in HCC. (d) Kaplan–Meier plots comparing the disease-free survival rates with high expression and low expression in HCC.

To further identify key genes with more reliable support for their involvement in the pathogenesis of HCC from the key module, GEPIA was used to analyze the transcript expression of the key module genes and their correlation with tumor stages, overall survival, and disease-free survival as derived from TCGA database. The statistical samples included 50 normal samples and 369 hepatocellular carcinoma samples. As shown in Table 4, UBE2L3 was upregulated in HCC and showed a significant positive correlation with tumor stage and negative correlations with OS and DFS (Figure 8). It was identified as a key gene in the pathogenesis of HCC.

### 3.7. Construction of circRNA-miRNA-Key Gene Network and qRT-PCR

**Figure 9:**
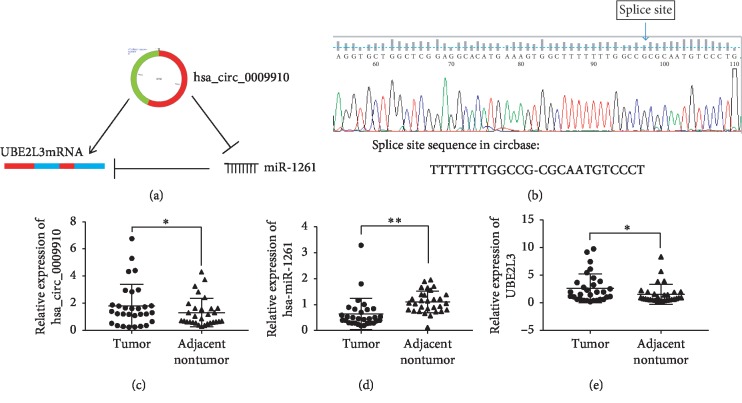
Regulatory axis construction and qPCR validation. (a) hsa_circ_0009910–miR-1261–UBE2L3 regulatory axis. (b) Sanger sequencing for circRNA primer verification. (c–e) Validation of expression of hsa_circ_0009910–miR-1261–UBE2L3 axis members in 30 pairs of HCC and adjacent nontumor tissues by qRT-PCR. ^*∗*^*P* < 0.05, ^*∗∗*^*P* < 0.01.

UBE2L3 was selected as a key gene from the key module; then, the hsa_circ_0009910–miR-1261–UBE2L3 axis was constructed based on the former ceRNA network. The expression levels of the axis members were examined by qRT-PCR in 30 pairs of samples including HCC tissues and adjacent nontumor tissues. The results showed that the relative expression levels of hsa_circ_0009910 and UBE2L3 were 1.812 ± 0.291-fold and 2.41 ± 0.4792-fold upregulated and that of miR-1261 was 0.634 ± 0.1109-fold downregulated in HCC versus adjacent nontumor tissues (Figure 9).

## 4. Discussion

As a result of the development of high-throughput RNA sequencing and novel biochemical/computational biology methods, an increasing number of studies have shown the importance of circRNAs in the initiation and development of various diseases, including malignant cancers [25]. circRNAs can often serve as biomarkers for diagnosis and prognosis because of their diversity and tissue-specific expression as well as their stability based on the covalently closed loop structures [26]. In HCC in particular, substantial evidence has been accumulated to prove the critical roles of circRNAs. With the intensification of research on the mechanisms of circRNA activity, their function of acting as miRNA sponges in the process of tumor development has been proven [5, 13]. Although the roles of some circRNAs in HCC have been identified [27, 28], it is suggested that many more potentially significant circRNAs have yet to be identified, which requires further exploration and research.

In the present study, using multiple cohort profile datasets and integrated bioinformatic analysis, a total of 11 differentially expressed circRNAs and 315 differentially expressed miRNAs were screened from four GEO databases. Based on the mechanism of conserved endogenous circRNAs harboring abundant miRNA binding sites to act as miRNA sponges and to function as ceRNAs to regulate gene expression [29–31], a total of 15 DEMs were screened out to interact with the candidate circRNAs and 560 mRNAs were selected as potential target genes of them. To further speculate on the function of the ceRNA network, functional annotation and pathway analysis of the target genes were performed. The results of the GO and KEGG pathway enrichment analyses suggested that the target genes were significantly enriched in different cancer-related functions and pathways. To identify key module in the target genes, a PPI network was constructed and combined with hub gene and module analyses, and module 2 was identified as a key module. Its genes were found to be particularly associated with protein ubiquitination (in Biological Process terms) and ubiquitin-mediated proteolysis (in the KEGG pathway analysis). They were also significantly related to the cancer process. The module genes were further analyzed based on the TCGA database and UBE2L3 was identified as a key gene associated with the pathogenesis of HCC. Then, the hsa_circ_0009910–hsa-miR-1261–UBE2L3 regulatory axis was constructed and its expression was verified by qPCR. In the study, the expression of hsa_circ_0009910 and UBE2L3 was upregulated and that of hsa-miR-1261 was downregulated in HCC, which is consistent with the theory of ceRNA [29].

It is well known that circRNA-mediated ceRNA pathways are essential for multiple functions, with the target mRNA determining their function based on ceRNA theory [32]. In the current study, the functions of the target genes and the key module were all particularly associated with ubiquitin-mediated proteolysis, as revealed by the KEGG pathway analysis. Protein ubiquitination is an important posttranslational mechanism for regulating the activity and levels of proteins in various conditions, including cancer [33]. In recent years, many studies have indicated the roles of protein ubiquitination and ubiquitin-mediated proteolysis in tumorigenesis, involved in regulating cell cycle progression, apoptotic factors, cancer metastasis, and the tumor-associated microenvironment. Disrupted regulation of protein ubiquitination may be one of the triggers for tumor development [34]. Therefore, the circRNAs in the ceRNA network may also play a tumor regulatory role through ubiquitin-mediated proteolysis.

Based on the integrated bioinformatic analysis, UBE2L3 was finally identified as a key gene among the target genes. It is one of the 38 ubiquitin-conjugating enzymes (E2) and participates in the ubiquitin transfer pathway and protein degradation [35]. It was previously observed that UBE2L3 expression may play an important role in the pathobiology of HCC and be expressed more highly in HCC samples than in normal tissues; in addition, increased expression of UBE2L3 is associated with the development of HCC, which matches our results. We also found that its expression was positively correlated with tumor stage through TCGA database. Liu et al. found that UBE2L3 was ubiquitously expressed in all cell lines, but it was expressed more highly in the strongly metastatic types. Upon UBE2L3's overexpression in the SNU-423 cell line, cellular proliferation and migration were enhanced, while they were inhibited upon its knockdown in QGY-7703. Further study found that UBE2L3 may degrade CDKN2B and CLDN1 [36]. Therefore, UBE2L3 may be an important oncogene in the development of HCC, but the upstream mechanism associated with it has not previously been reported.

It is widely recognized that miRNA-mediated pathways play roles in tumorigenesis, including cell proliferation, migration, and apoptosis. In the ceRNA network established in this study, 15 differentially expressed miRNAs were included, including 8 upregulated and 7 downregulated ones in tumor samples. They mediated the link between circRNA and target genes. Hsa-miR-1261, upstream of UBE2L3, was downregulated in HCC; its role in tumorigenesis has been reported in several different tumor types. For example, Zhang et al. reported that miR-1261 could regulate the expression of circ-PTPRZ1/PAK1 and inhibit the proliferation and invasion and promote the apoptosis of glioma cells [37]. Moreover, Hong et al. reported that miR-1261 was downregulated in thyroid cancer cells and plays an inhibitory role against proliferation and invasion [38]. The role of miR-1261 as identified in the present study appears to be similar to the results of previous research, but further verification of this is required.

Hsa_circ_0009910 was one of the 11 commonly upregulated circRNAs in the three datasets. Its role in tumors has been tentatively explored in recent years. For example, Ping et al. reported that circ_0009910 was significantly upregulated in acute myeloid leukemia patients compared with its level in iron deficiency anemia patients, and its high expression was predictive of poor prognosis; moreover, its silencing could inhibit cell proliferation and induce apoptosis through increasing miR-20a-5p [39]. Its procancer effects have also been verified in gastric cancer and osteosarcoma [40, 41], but its expression and its correlation with prognosis in HCC have never been reported.

In our study, hsa_circ_0009910 and UBE2L3 were confirmed to be highly expressed and miR-1261 was expressed at a low level by qPCR in 30 pairs of samples from HCC patients, including tumor tissues and adjacent nontumor tissues. Combined with bioinformatic prediction, it is preliminarily suggested that hsa_circ_0009910–miR-1261–UBE2L3 axis may exhibit a regulatory relationship in the pathogenesis of HCC. Hsa_circ_0009910 may be a novel molecule involved in the carcinogenesis of HCC. However, our study is based on bioinformatic analysis, so further experiments are needed to confirm the conclusions made here.

## Figures and Tables

**Table 1:** Information on the three circRNA microarrays and one miRNA microarray.

Series	Type of microarray	Tumor	Nontumor	Platforms	Reference
GSE78520	circRNA profile1	3	3	GPL19978	—
GSE97332	circRNA profile2	7	7	GPL19978	[19]
GSE94508	circRNA profile3	5	5	GPL19978	[20]
GSE64632	miRNA profile	3	3	GLP18116	[21]

**Table 2:** The differentially expressed circRNAs and miRNAs from the downloaded expression profiles.

Dataset	Upregulation	Downregulation	Total
GSE78520	128	17	145
GSE97332	440	427	867
GSE94508	200	337	537
GSE64632	227	88	315

**Table 3:** Basic characteristics of differential expressed circRNAs.

Circbase ID	Spot_ID	Position	Spliced length	Strand	Best transcript	Gene symbol	Regulation
hsa_circ_0072088	hsa_circRNA_103809	chr5 : 32379220-32388780	693	−	NM_016107	ZFR	Up
hsa_circ_0051732	hsa_circRNA_102587	chr19 : 48660270-48660397	127	−	NM_000234	LIG1	Up
hsa_circ_0005397	hsa_circRNA_102034	chr17 : 30500849-30503232	233	+	NM_001033568	RHOT1	Up
hsa_circ_0000673	hsa_circRNA_101707	chr16 : 11940357-11940700	251		NM_015659	RSL1D1	Up
hsa_circ_0001338	hsa_circRNA_001416	chr3 : 128824688-128825122	434	−	NM_001204883	RAB43	Up
hsa_circ_0003945	hsa_circRNA_104760	chr9 : 33953282-33956144	258	−	NM_018449	UBAP2	Up
hsa_circ_0027478	hsa_circRNA_101094	chr12 : 69109406-69125499	1029	+	NM_020401	NUP107	Up
hsa_circ_0092283	hsa_circRNA_400071	chr22 : 36681395-36681695	300	−	NM_002473	MYH9	Up
hsa_circ_0003923	hsa_circRNA_102954	chr2 : 238933982-238940895	162	+	NM_080678	UBE2F	Up
hsa_circ_0009910	hsa_circRNA_100053	chr1 : 12049221-12052747	315	+	NM_014874	MFN2	Up
hsa_circ_0001901	hsa_circRNA_000996	chr9 : 138773785-138774005	220	−	NM_015447	CAMSAP1	Up

**Table 4:** TCGA analysis of key module genes.

Module gene	Hub gene (top 20)	Upregulate	Stage		Overall survival		Disease-free survival	
			*F* value	*P* value	HR	*P* value	HR	*P* value
VHL	Yes	–	2.4	0.0677	1.4	0.083	1.5	0.015^*∗*^
CDC26	No	–	2.4	0.04^*∗*^	1.5	0.025^*∗*^	1.6	0.0031^*∗∗*^
ANAPC11	No	Tumor^*∗*^	2.61	0.513	1.3	0.13	1.3	0.084
UBE2L3	Yes	Tumor^*∗*^	4.5	0.00409^*∗*^	1.8	0.0011^*∗∗*^	1.5	0.0077^*∗∗*^
SOCS3	Yes	Normal^*∗*^	0.651	0.583	1	0.92	0.95	0.72
NEDD8	Yes	Tumor^*∗*^	1.13	0.336	1.3	0.11	1.6	0.0041^*∗∗*^
UBE2G1	Yes	–	1.01	0.39	1.1	0.67	0.9	0.49
HECTD2	No	–	1.76	0.155	1.8	0.0011^*∗∗*^	1.5	0.0077^*∗∗*^

## Data Availability

Expression profile of GSE78520, GSE97332, GSE94508, and GSE64632 in the manuscript was downloaded from NCBI-GEO (https://www.ncbi.nlm.nih.gov/gds).

## Abbreviations

HCC: Hepatocellular carcinoma
miRNA: MicroRNA
circRNA: Circular RNA
MRE: MicroRNA response element
GEO: Gene expression omnibus
DECs: Differentially expressed circRNAs
DEMs: Differentially expressed miRNAs
GO: Gene ontology
KEGG: Kyoto encyclopedia of genes and genomes
PPI: Protein-protein interaction
TCGA: The cancer genome atlas.
